# Increased functional connectivity in the right dorsal auditory stream after a full year of piano training in healthy older adults

**DOI:** 10.1038/s41598-023-46513-1

**Published:** 2023-11-15

**Authors:** Kristin Jünemann, Anna Engels, Damien Marie, Florian Worschech, Daniel S. Scholz, Frédéric Grouiller, Matthias Kliegel, Dimitri Van De Ville, Eckart Altenmüller, Tillmann H. C. Krüger, Clara E. James, Christopher Sinke

**Affiliations:** 1 Division of Clinical Psychology & Sexual Medicine, Department of Psychiatry, Social Psychiatry and Psychotherapy, Hannover Medical School, Hannover, Germany; 2 Center for Systems Neuroscience, Hannover, Germany; 3 Geneva Musical Minds Lab, Geneva School of Health Sciences, University of Applied Sciences and Arts Western Switzerland (HES-SO), Geneva, Switzerland; 4 Faculty of Psychology and Educational Sciences, University of Geneva, Geneva, Switzerland; 5 CIBM Center for Biomedical Imaging, MRI UNIGE, University of Geneva, Geneva, Switzerland; 6 Institute of Music Physiology and Musicians′ Medicine, Hannover University of Music, Drama and Media, Hannover, Germany; 7 Institute of Medical Psychology, University of Lübeck, Lübeck, Germany; 8 Department of Musicians’ Health, University of Music Lübeck, Lübeck, Germany; 9 Swiss Center for Affective Sciences, University of Geneva, Geneva, Switzerland; 10 Center for the Interdisciplinary Study of Gerontology and Vulnerability, University of Geneva, Geneva, Switzerland; 11 Neuro-X Institute, École Polytechnique Fédérale de Lausanne (EPFL), Geneva, Switzerland; 12 Department of Radiology and Medical Informatics, University of Geneva, Geneva, Switzerland

**Keywords:** Neuroscience, Cognitive neuroscience

## Abstract

Learning to play an instrument at an advanced age may help to counteract or slow down age-related cognitive decline. However, studies investigating the neural underpinnings of these effects are still scarce. One way to investigate the effects of brain plasticity is using resting-state functional connectivity (FC). The current study compared the effects of learning to play the piano (PP) against participating in music listening/musical culture (MC) lessons on FC in 109 healthy older adults. Participants underwent resting-state functional magnetic resonance imaging at three time points: at baseline, and after 6 and 12 months of interventions. Analyses revealed piano training-specific FC changes after 12 months of training. These include FC increase between right Heschl’s gyrus (HG), and other right dorsal auditory stream regions. In addition, PP showed an increased anticorrelation between right HG and dorsal posterior cingulate cortex and FC increase between the right motor hand area and a bilateral network of predominantly motor-related brain regions, which positively correlated with fine motor dexterity improvements. We suggest to interpret those results as increased network efficiency for auditory-motor integration. The fact that functional neuroplasticity can be induced by piano training in healthy older adults opens new pathways to countervail age related decline.

## Introduction

Making music is a multimodal task that requires not only auditory-sensory-motor integration and emotional processing, but also higher order cognitive functions^1,2^. These cognitive functions, which include processing speed, attention, executive functioning, as well as working memory are all known to naturally decline with age^3^.

Therefore, to fully grasp brain processes underlying the production of music and its potential effects on aging, it is important to study the interaction between the different brain areas involved in these processes. One way to quantify these interactions is using functional connectivity (FC) by calculating and comparing the temporal correlation between different parts of the brain. FC can be measured using resting-state functional Magnetic Resonance Imaging (rs-fMRI). Brain regions that are functionally related, share similar spontaneous low-frequency fluctuations during rest^4^. This way, distinct functionally connected neural networks have been identified in the human brain, such as primary processing networks, which include the sensorimotor network (SMN), and the auditory network (AN), but also higher-order networks, like the executive control network (ECN, involved in goal-directed behavior, attention and working memory) and the default mode network (DMN)^5^. The DMN is unique as it shows a high level of activity at rest, but lower levels of activity when performing a task^6^. Most interestingly, it is the most consistently found network to be affected by aging (for reviews see e.g. ref.^5,7^). Another higher-order network that has been associated with non-pathological cognitive ageing in a large scale study is the cingulo-opercular network (CON), involved in cognitive control and goal-directed behaviour^8^. Often the CON network is also referenced as the salience network. For other networks, especially the primary processing networks, findings are more diverse. However, most studies find decreases in the AN and no change or decreases in the SMN in older adults^5^.

When investigating the effects of playing an instrument, typically musicians and non-musicians are compared. Here, the most consistent finding among studies is a higher FC between auditory and motor regions in musicians^9–13^. Interestingly, the largest study to date^14^ found increased FC within the AN and between bilateral superior and middle temporal, inferior frontal, and inferior parietal regions, but not between auditory and motor regions in musicians compared to non-musicians. Thus, results are still too inconsistent to draw clear conclusions about the exact effects of musical training on functional brain plasticity. One main problem is that most studies used relatively small sample sizes, which not only leads to a reduced chance of finding a true effect but also increases the probability that significant results do not reflect a true effect^15^.

Few studies investigated the effect of musical training on FC in longitudinal settings and those who did also used relatively small sample sizes. One study^16^ investigated eight weeks of drum training in a group of 15 young (16–19 years old) participants compared to a passive control group and identified a significant increase in FC between auditory and motor regions as well as between auditory and parietal regions in the drum group but not in the control group over time. Another study conducted over 24 weeks of piano training found increased FC within the SMN and between auditory-motor regions in 29 young adults compared to a passive control group^17^. However, these effects receded after 12 weeks of no training^17^. While these studies used younger populations to study the effect of musical practice, learning to play an instrument has also become a target intervention to slow down or counteract age-related cognitive decline in recent years^18–20^. Although these studies show promising results, they lack statistical power (between 8 and 16 participants in the intervention groups) and they did not use neuroimaging methods to investigate the underlying neural adaptions induced by the training.

Yet, important factors in the aging process of the human brain are maladaptive changes of its FC, most often resulting in reduced cognitive performance^5^. These include reduced FC within and between networks, but also increased FC, which is usually interpreted as a compensatory mechanism^21–25^. Based on the above-mentioned studies, musical training might be a promising tool to promote successful aging, potentially altering connectivity between brain areas, which in consequence may lead to preservation or even improvement of cognitive abilities^26^. Especially since Rogenmoser et al.^27^ have suggested, based on structural data, that music as a leisure activity can have an age-decelerating effect on the brain.

A recent publication by our consortium^28^ investigated the effect of six months of piano practice (PP) on white matter structural connectivity in healthy older adults as part of a larger randomized controlled trial (RCT). Analyses revealed a stabilizing effect of PP on microstructure of the fornix (a main output tract of the hippocampus), but no significant (Bonferroni-corrected) effects in auditory and motor-related tracts. However, PP participants still advanced in their playing abilities. While these improvements could not be explained by structural connectivity changes, here we investigate whether FC changes may underlie these improvements, using the same study sample. In addition, we are interested in whether piano training also influences FC of the DMN, which is known to be affected by aging and other primary processing networks, where the exact impact of ageing is still unknown^5^. The current study aims to investigate FC changes after 6 and 12 months of PP compared to an active control group participating in music listening/musical culture (MC) lessons in a large older adults’ sample. Specifically, we use seed-to-whole-brain FC analysis with the following 8 seeds that have been implicated in previous studies on musical expertise and aging: Heschl’s gyrus (HG, belonging to the AN)^29^, motor hand area (M1H, belonging to the SMN)^30^, hippocampal formation (HPF, belonging to the DMN)^31^, and inferior frontal gyrus (IFG, belonging to the ECN)^32^.

The HG is affected by musicians’ status in a large number of studies^33–38^, with changes being related to music proficiency^33,34^. The HG, as part of the auditory cortex, is anatomically linked to the primary motor cortex and the IFG via the arcuate fasciculus^39^. The IFG has been shown to be involved in music processing^40,41^ and the generation and selection of motor sequences^42^. Due to its involvement in working memory processes^43^ the IFG represents an interesting seed region. Improvements in working memory have been shown after six months of piano training in healthy older adults^18,20^, although working memory is particularly affected by age^3^. As playing the piano specifically engages fine motor control of hands and fingers, we selected a seed located at the M1H^44^. In an earlier publication of this study we showed that in comparison with the MC group, first, practicing piano resulted in greater improvements in manual dexterity and second, that unimanual dexterity and grey matter volume of the contralateral M1 changed together during the second semester of training^45^. Further, fine motor control is known to deteriorate with advanced age^46,47^. Another region of interest is the HPF, not only because of its involvement in aging and cognitive decline^48^, but also because of its involvement with music-evoked emotions^49^ and higher-order pitch processing^50–52^.

Each seed was investigated separately for the left and right hemispheres. We hypothesize that learning to play the piano primarily leads to FC increase between auditory and motor regions as found in previous studies^9–13,16,17^, verifying the association of those FC changes with behavioural measures and piano practice progress, closely linked to piano training, so-called near-transfer, primarily expressed in auditory and motor tasks. In addition, we hypothesize that learning to play the piano has the potential to counteract or slow down age-related FC changes, including increased FC in and between the SMN and AN. Considering the limited literature on the effects of musical instrument playing on the ageing DMN and ECN, we included these networks for exploratory analyses, hypothesizing that PP could have a beneficial effect counteracting age-related changes in these networks.

## Materials and methods

The study was reviewed and approved by the ethics committee of Hannover Medical School as well as the cantonal ethics committee Geneva. All participants gave written informed consent before participation and all experiments were compliant with the Declaration of Helsinki.

### Participants and study design

A total of 155 subjects (62–78, mean age = 69.7 years, 92 females) were recruited for the current RCT in Hannover (Germany, 92 participants) and Geneva (Switzerland, 63 participants). Participants had to be in good overall health, right handed^53^, retired, and non-reliant on hearing-aids. Furthermore, included participants should not have received more than 6 months of formal musical training outside the school curriculum during their lifetime. The general sophistication scale of the Goldsmiths Musical Sophistication Index (Gold-MSI) was assessed to measure their musical sophistication at baseline (minimum/maximum achievable score = 18/128)^54^. The cognitive telephone screening instrument (COGTEL)^55,56^ was used to assess cognitive functioning and avoid the inclusion of participants with early-stage dementia. As this test was specifically developed for older adults, it provides a global measure of cognition, based on several memory and executive function subtests. A cut-off value of below 10 was set a priori based on the original publications^55,56^, however each participant in the current study reached a score of at least 15. The participants were randomly assigned to one of the two intervention groups: PP or MC. In order to obtain homogeneous groups, age, gender, education level, and COGTEL score were taken into account to achieve equal distributions of these factors across both groups. Participants only learned of their group assignment after baseline testing. Both groups received one 60 min lesson per week for one year and were instructed to do homework daily for at least 30 min. PP lessons were taught in dyads whereas MC lessons took place with 4–6 participants. The lessons were designed and regularly supervised by two music pedagogy professors. Twenty-four teachers were involved who all were professional musicians with at least a bachelor’s degree. Participants in the PP group learned to play the piano with both hands (separately at first), how to read a musical score, and played different styles of music. MC lessons focused on active and analytical listening to music of different epochs and genres, learning about music history and musical styles, and information on basic formal and structural properties. The course aimed to create general enjoyment and appreciation of music, however, it excluded any active music-making (e.g. singing), or other music-related movements (e.g. clapping). More details about the study design can be found in James et al.^57^ and in the supplementary material of Worschech et al.^58^. To monitor the participants’ adherence to the study protocol, daily homework logs were collected. Adherence was based on self-report from the participants on how much they practiced daily at home, which had to be put into a training diary and collected regularly. Participation was assessed by the teachers after 6 and 12 months. In addition, the teachers followed the participants’ progress to make sure they did indeed practice at home. We observed a solid adherence to the interventions in all participants.

Participants underwent testing before the start of the interventions (T0), after 6 months of lessons (T1), and after 12 months of lessons (T2). Due to the COVID-19 outbreak and local restrictions, there were delays in testing at T2 for the majority of participants. Although some participants had to finish their courses online, all included participants had at least 46 lessons when T2 testing took place. To factor in the delays in testing, all statistical models include the time between measurements as a covariate.

Of the 155 participants, 120 completed MRI scanning at all 3 time points. Of these, 4 had to be excluded from the analyses because of artefacts in the T1 image, one participant was excluded because of too much atrophy and myelin degeneration, 6 participants showed excessive head movement and had less than 150 of 460 remaining volumes in the functional scan after head-motion scrubbing. This resulted in a total of 109 participants who were included in the final analyses (53 in the MC group and 56 in the PP group).

### Image acquisition

MR images were acquired on 3.0 T Siemens MRI scanners (Hannover: Magnetom Skyra, Geneva: Magnetom Tim Trio; Siemens, Erlangen, Germany) using 32-channel head coils. Both study sites used the same scanning parameters. For the resting-state fMRI images, the following echo planar imaging (EPI) sequence was used: voxel size = 2.5 mm isotropic, FoV = 210 × 210 × 135 mm^3^, multiband acceleration factor = 3, repetition time (TR) = 1350 ms, echo time (TE) = 31.6 ms, and 460 volumes. The scan lasted 10.31 min and participants were instructed to focus on a fixation cross in the middle of the screen during scanning. Additionally, a high-resolution T1-weighted structural image was acquired, using the following MP2RAGE sequence^59^: voxel size = 1 mm isotropic, FoV = 256 × 240 × 176 mm^3^, TR = 5000 ms, TE = 2.98 ms, flip-angle 1 = 4°, flip-angle 2 = 5°, and 176 slices. To account for potential site effects and different MRI scanners, this factor was included as a covariate of no interest in all statistical analyses. For noise reduction during scanning, subjects wore Comply™ Canal Tips with an average noise reduction of 29 dB.

### Preprocessing

The data was analyzed using the Data Processing Assistant for Resting-State Toolbox (DPARSFA, http://rfmri.org/DPARSF)^60^ and SPM12 (Wellcome Trust Centre for Neuroimaging, London, England). Preprocessing followed the recommended standard DPARSFA protocol.

In a first step, the first five image volumes of the functional images were discarded to account for initial signal instability. The remaining images were then slice-timing corrected and realigned to a mean image. Voxel-specific head motion parameters were calculated. Then structural images were co-registered to the functional images and segmented into white matter (WM), grey matter (GM), and cerebrospinal fluid (CSF) maps using DARTEL^61^. The Friston 24-parameter model^62^ was used to regress out motion artefacts from the realigned images. In addition, WM, CSF, and global signal were regressed out. Images were then normalized to MNI space using DARTEL and temporally bandpass filtered (0.01–0.10 Hz). In the next step, head motion scrubbing was performed, which removed time points with a framewise displacement of more than 0.4 mm^63^. In the last step, images were smoothed with a Gaussian kernel of 7 mm^3^ (full width at half maximum).

### Seed-based functional connectivity analysis

Calculation of FC was carried out with a seed-based approach, using the DPARSFA toolbox^60^. Here, the time course of each seed (i.e. the averaged time courses of all voxels within this region) was correlated pairwise with the time course of all other voxels within the brains’ GM (Pearson’s correlations). Then individual’s correlation (r) values were normalized by means of Fisher’s *z* transformation and evaluated using an SPM second level model. Difference images for T1-T0 and T2-T0 were calculated and entered into a General Linear Model to assess changes after 6 and 12 months respectively. Anatomical locations of the significant clusters were identified using the Automated anatomical labelling atlas (AAL3)^64^.

### Seed regions

As outlined in the introduction, eight different seeds were selected based on a recent study by our consortium^28^, their relevance in music making/musical processing, aging brain literature, and their susceptibility to training-induced changes.

**Figure 1:**
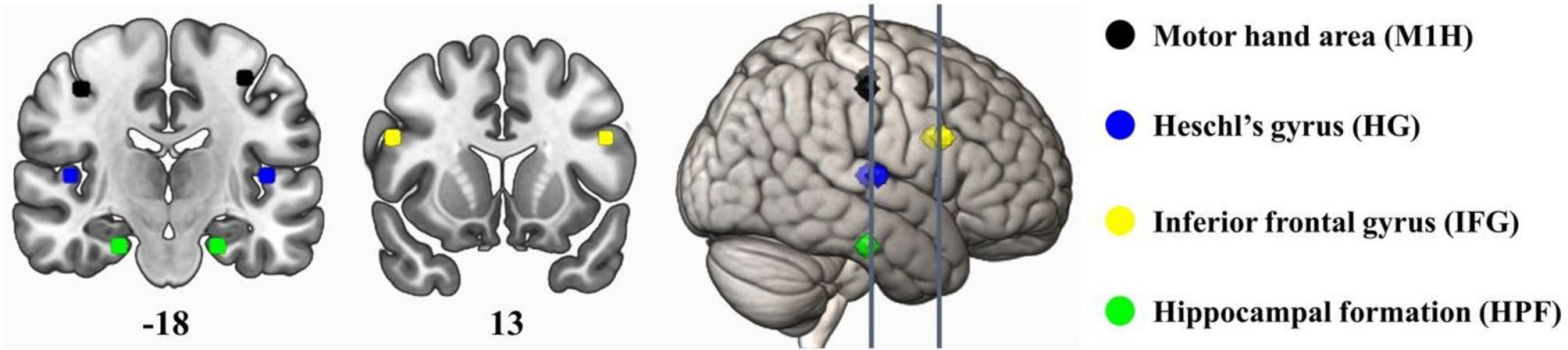
Seed regions visualized on MNI template. Coronal slices are displayed in radiological convention (left = right hemisphere, right = left hemisphere).

Peak coordinates for the seeds were selected based on existing literature and a mask for each seed was created using a 5 mm radius around the peak coordinates. Table 1 summarizes the selected seeds, their peak MNI coordinates and the reference the coordinates are based on. Locations of the selected seeds are visualized in Fig. 1.

**Table 1:** Seed regions.

Seed	Hemisphere					
	Left			Right		
	x	y	z	x	y	z
Heschl’s gyrus (HG)^65^	− 46	− 20	8	46	− 16	8
Motor hand area (M1H)^44^	− 36	− 21	52	39	− 19	48
Hippocampal formation (HPF)^31^	− 22	− 20	− 26	22	− 20	− 26
Inferior frontal gyrus (IFG)^43^	− 48	10	26	50	14	24

Peak coordinates for the functional connectivity analysis in MNI space, based on existing literature.

### Behavioral tests

As this analysis is focusing on resting-state FC, only tests that are relevant for the statistically significant FC changes are explained in detail below. As explicated in the hypothesis, the focus of this analysis is on the acquisition of a motor skill. Thus, for the evaluation of near transfer effects of the acquisition only motor tasks were evaluated. For a full list of administered tests in the study, please refer to James et al.^57^.

#### MIDI-based scale analysis

An adaption of the MIDI-based scale analysis (MSA)^66^ was used in both groups to measure improvements in piano playing. This measure has previously been shown to be an indicator of pianistic expertise^67^. Participants were instructed to play sequences of 15 five-tone range scales (C-G) in both playing directions with the right hand, using the following fingering: 1-2-3-4-5-4-3-2-1-2-3-4-5-4-3-2-1 (1 = thumb, 5 = little finger). The outcome measure was the mean standard deviation of the time between the onsets of two subsequent notes (IOI = inter-onset interval). The desired IOI was set to 790 ms and paced by a metronome.

As we considered this task too difficult and thus frustrating to perform with the left hand for the MC group at T1 and T2, this test was performed with the right hand only.

#### Purdue pegboard

The Purdue Pegboard Test (PT)^68^ was used to measure fine motor dexterity and gross movements of the hands, fingers and arms. The PT consists of three sub tests that all measure gross hand and arm control: right hand (PT-RH), left hand (PT-LH), and both hands (PT-BH, also measuring bimanual coordination). These tests take 30 s each and consist of placing as many pins as possible in a vertical row with the specified hand (PT-RH and PT-LH) or both hands at the same time (PT-BH).

Moreover, there is another assembly subtest measuring fine finger/ fingertip dexterity and bimanual coordination (PT-A). In this test, participants have 60 s to assemble a small tower consisting of a pin, a washer, a collar, and another washer. Final scores consist of the total number of pins placed/objects assembled within the given time.

### Statistical analysis

#### Group-level

In a first step, only baseline measurements were taken into account to assess whether the two groups differed in FC before the start of the interventions. Group differences at baseline were assessed with two-sample *t* tests entered into separate General Linear Models (GLM) for the eight seeds of interest in SPM12, including site, age at the start of the intervention (demeaned), and gender as nuisance covariates.

In a next step, to assess training-related changes, difference images were calculated between T0 and T1 as well as T0 and T2 and also entered into a GLM, with site, age at the start of the intervention (demeaned), gender, and time between scans (demeaned) as nuisance covariates. Separate GLMs were created for each seed for six and twelve months’ data. To measure FC changes over time, one-sample *t* tests including the full sample (both groups combined) were calculated (PP^+^  + MC^+^ for increased FC over time and PP^–^ + MC^–^ for decreased FC over time).

Group differences (over time) between the two intervention groups were assessed with a two-sample *t* test (MC > PP and PP > MC). To account for the amount of voxels in the whole brain analysis, we used FWE correction at cluster level, and lowered the significance threshold further, to account for the eight seed regions, resulting in a significance threshold for each seed region of p_FWE_ < 0.05/8 = 0.00625.

In a last step, it was important to know whether the seeds and cluster regions were positively or negatively correlated (i.e. anticorrelated) at baseline to distinguish between different FC changes over time. A FC increase over time could for example mean a higher positive correlation or a decreased anticorrelation. For this purpose, we created binary masks from the significant clusters and used these to extract the baseline correlations from the T0 images for the respective seed regions.

Further statistical analyses were performed in IBM SPSS Statistics version 27 (IBM Corp., Armonk, NY, USA). As difference images were entered into the GLMs, significant effects do not quantify where this difference originates from. Therefore, the mean value over the significant cluster was extracted and compared against 0 for each group using a one-sample *t* test. A non-significant test result implicated no change over time for this group, while a significantly higher mean would quantify an FC increase and a lower mean a significant FC decrease over time. Due to the number of tests (12 in total), Bonferroni correction was used, setting p < 0.0042 as the significance threshold.

#### Behavioral and correlational analyses

To keep in line with the FC analysis, we performed separate analyses for 6 and 12 months data. Hence, MSA was analyzed using two 2 × 2 (Group × Time) mixed-design ANOVAs, with group as a between-subject variable and time as a within-subject variable. Outliers with more than two SD from the mean were excluded from this analysis. This was the case for four participants. Eight additional participants with at least one missing value were excluded. Therefore, the final analysis on MSA comprised 45 MC and 52 PP participants. PT was analyzed separately for the four sub-tests, each with a 2 × 2 (Group × Time) mixed-design ANOVA. For correlation analyses, difference scores were calculated and correlated with extracted cluster FC changes (T1–T0, or T2–T0, depending on the outcome of the FC analysis). Correlations were only calculated if post-hoc analyses on FC revealed a significant FC change over time.

In total eight correlations were computed, therefore, the significance threshold was set to p < 0.00625.

## Results

### Demographic data (N = 109)

Both groups did not differ in respect to age, gender, education level, COGTEL score and Gold-MSI score (Table 2).

**Table 2:** Demographics (N = 109).

	Mean (sd)		Group comparison	
	PP	MC	Statistic	p
Age	69.28 (3.28)	69.51 (3.96)	t = 0.33	0.74
Males/females	25/31	24/29	χ^2^ = 0.005	0.95
Education	3.91 (1.38)	3.85 (1.41)	χ^2^ = 2.77	0.74
COGTEL score	31.44 (6.87)	32.46 (7.39)	t = 0.74	0.46
Gold-MSI score	49.68 (12.33)	49.77 (11.62)	t = 0.41	0.97

Group comparisons did not reveal any significant differences between both groups in age, gender, education level and COGTEL score. Group differences were calculated using a two-sample independent *t* test for age and COGTEL score, and a chi-squared test for gender and education. Education is a categorical variable with 6 levels (1 = primary school, 2 = middle school, 3 = high school, 4 = Bachelor’s degree, 5 = Master’s degree, 6 = doctorate degree).

*COGTEL* cognitive telephone screening instrument, *Gold*-*MSI* goldsmiths musical sophistication index, *PP* piano practice group, *MC* music listening/musical culture group, *sd* standard deviation.

### FC group differences at baseline

No significant group differences were detected at baseline.

### FC results—6 months

There were no significant effects after 6 months of training.

### FC results—12 months (T2)

No significant effects were found for the left HG, left M1H, right HPF, and left IFG seeds. The right IFG was the only seed region that showed a main effect of time. Across all participants, there was a significantly higher FC (p_FWE_ = 0.002, k (number of voxels in the significant cluster) = 310, T = 4.46) between the right IFG and the left angular gyrus after twelve months of interventions. For that seed, no significant effect of group could be detected. The right HG, right M1H and left HPF all revealed significant FC group differences over time. The only FC seed which showed a significant MC > PP effect of group after 12 months was the right HG. These significant group results are described in more detail below.

**Figure 2:**
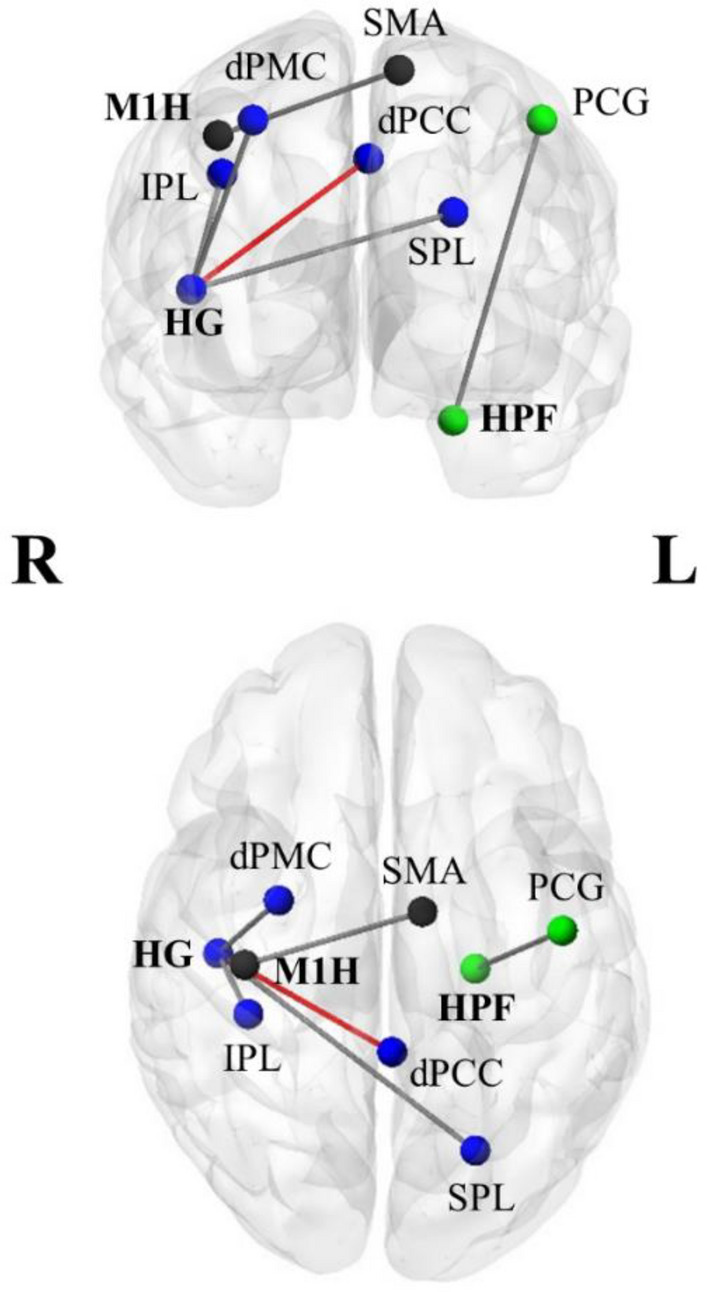
Results for the PP group after 12 months of intervention. Significant FC increases or decreases that were found for the PP group in comparison to the MC group. Gray edges indicate increased FC and red edges indicate reduced FC between regions after piano training. Seed regions are printed in bold. Blue nodes = results for HG (Heschl’s gyrus), green node = results for HPF (Hippocampal formation), Black nodes = results for M1H (Motor hand area). *IPL* inferior parietal lobule, *dPCC* dorsal posterior cingulate cortex, *dPMC* dorsal premotor cortex, *PCG* precentral gyrus, *SPL* superior parietal lobule, *R* right, *L* left. Images are displayed in radiological convention (left = right hemisphere, right = left hemisphere). The results were visualized with BrainNet viewer^75^.

A summary of all significant effects after 12 months of interventions are shown in Table 3. Additionally, results for the PP group after 12 months are displayed in Fig. 2.

**Table 3:** Summary of significant effects after 12 months of interventions.

Contrast	Post-hoc tests		k	Peak MNI coordinates			T	AAL3 cluster regions (amount of cluster voxels in region)	Baseline connectivity
	MC	PP		x	y	z			Mean (sd)
Seed: right Heschl’s gyrus (HG)									
MC > PP	=	↓	279	0	− 42	42	4.68	L midcingulate cortex (dPCC, 40%)*, R midcingulate cortex (dPCC, 38%), R precuneus (12%)	− 0.072 (0.11)
PP > MC	=	↑	594	− 22	− 68	28	5.59	L superior parietal lobule (51%), L superior occipital gyrus (19%)*, L inferior parietal lobule (13%), L middle occipital gyrus (6%)	− 0.078 (0.11)
	=	↑	288	30	− 2	52	4.88	R precentral gyrus (35%), R superior frontal gyrus (dPMC, 35%), R middle frontal gyrus (vPMC, 10%)*	0.084 (0.12)
	=	↑	360	38	− 32	38	4.35	R superior parietal lobule (40%), R postcentral gyrus (22%), R inferior parietal lobule (17%), R supramarginal gyrus (8%)*	0.062 (0.14)
Seed: right motor hand area (M1H)									
PP > MC	=	↑	2880	− 8	− 5	65	5.31	R postcentral gyrus (25%), R precentral gyrus (14%), R superior frontal gyrus (dPMC, 10%), R SMA (10%), L SMA (9%)*, R superior parietal lobule (9%), R supramarginal gyrus (5%), L precentral gyrus (3%), L superior frontal gyrus (dPMC, 3%)	0.515 (0.16)
Seed: left hippocampal formation (HPF)									
PP > MC	↓	↑	208	− 45	− 10	52	5.32	L precentral gyrus (59%), L postcentral gyrus (31%)*	− 0.102 (0.10)
Seed: right inferior frontal gyrus (IFG)									
PP^+^ + MC^+^	↑	↑	310	− 40	− 72	42	4.46	L angular gyrus (57%)*, L inferior parietal lobule (10%)	− 0.201 (0.23)

Significant clusters were identified using the automated anatomical labelling 3 atlas (AAL3)^64^. Only identified regions that included more than 25 voxels are mentioned. Peak activations are marked with an asterisk (*).

*k* number of voxels in the significant cluster, *MC* music listening/musical culture group, *PP* piano practice group, *dPCC* dorsal posterior cingulate cortex, *dPMC* dorsal premotor cortex, *vPMC* ventral premotor cortex, *SMA* supplementary motor area, *L* left, *R* right.

↓ significant FC decrease over time, ↑ significant FC increase over time, = no significant change over time.

#### Right Heschl’s gyrus—T2 results, MC > PP

**Figure 3:**
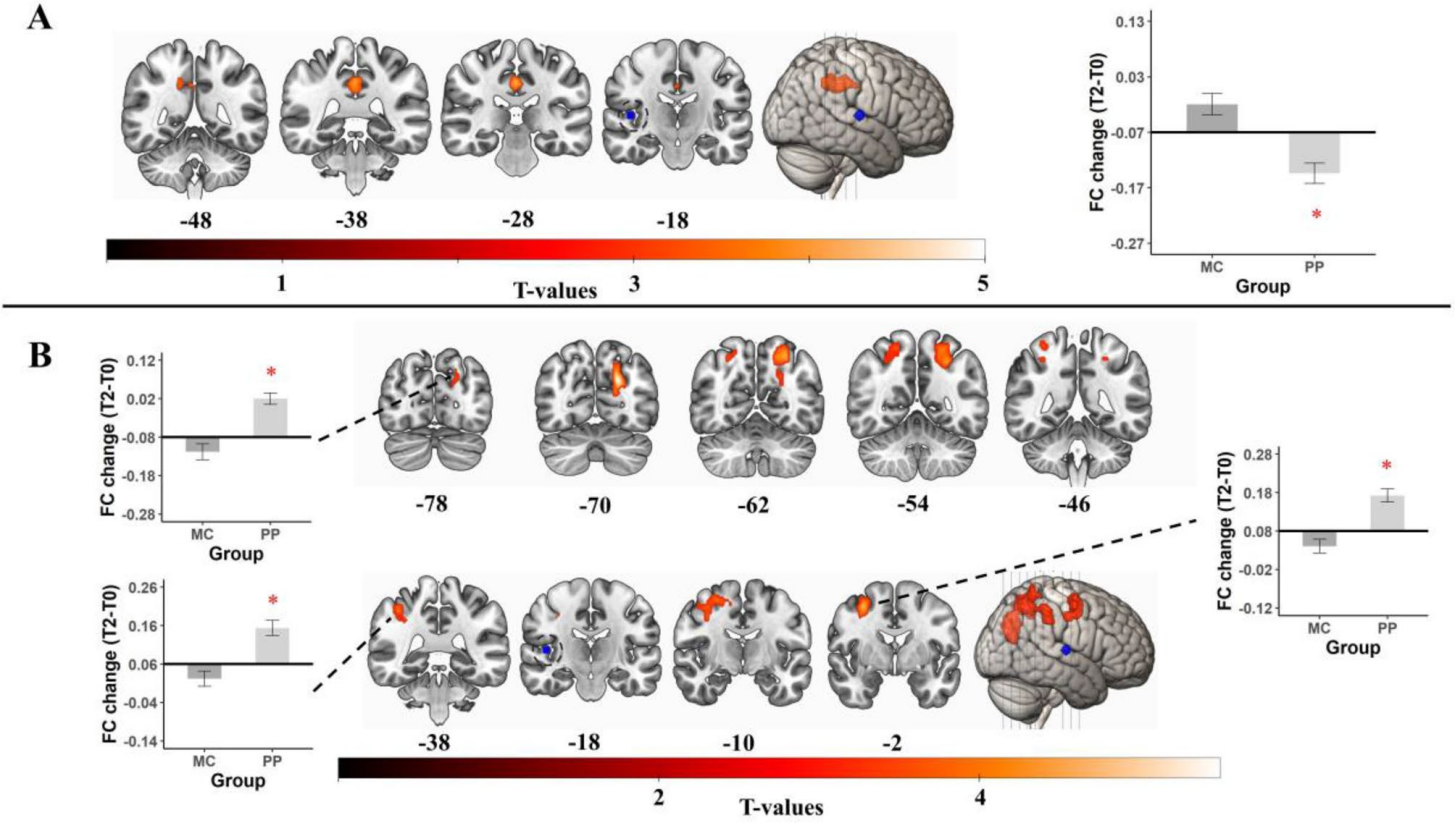
Results for right HG seed region. Regions that show a significantly MC > PP (**A**) and PP > MC (**B**) group difference after 12 months of interventions. Post-hoc *t* tests revealed that only the PP group showed a significant Bonferroni-corrected difference to 0, indicating a FC change over the 12 months of training in the PP group and no change in FC for the MC group. The mean values for each group are displayed in relation to the baseline correlation as bar plots next to the respective cluster. Bold line represents the baseline correlation. Error bars represent the standard mean error. Red asterisk (*) depict significant post-hoc t-tests. *MC* music listening/musical culture group, *PP* piano practice group, *FC* functional connectivity, *T0* baseline measurement, *T2* 12 months measurement. The seed location is shown in blue and circled. Coronal slices are displayed in radiological convention (left = right hemisphere, right = left hemisphere).

For the **right** HG, analyses revealed a significant MC > PP effect of group (p_FWE_ = 0.002) after 12 months of interventions. FC differences were found between the **right** HG and midcingulate cortices (k = 279, T = 4.68, Fig. 3A). As the midcingulate cortices are very broadly defined in the AAL3 atlas, we parcellated it into distinct subregions. Our cluster lies in a region that is most often defined as the dorsal posterior cingulate cortex (dPCC)^69–73^. Post-hoc t-tests revealed no significant Bonferroni-corrected effects over time for the MC group (mean = 0.012, sd = 0.14, t(52) = 2.605, p = 0.012), but a significant FC decrease in the PP group (mean = − 0.075, sd = 0.138, t(55) = − 4.008, p < 0.001).

Further analysis revealed a negative correlation at baseline between the right HG and the significant cluster (mean = − 0.072, sd = 0.11). This indicates a higher anticorrelation over time in the PP group.

#### Right Heschl’s gyrus—T2 results, PP > MC

Further, for the **right** HG there were multiple significant PP > MC group differences after 12 months of intervention (Fig. 3B). The first significant cluster was found in the left superior parietal lobule/ left superior occipital gyrus (p_FWE_ < 0.001, k = 594, T = 5.59). Post-hoc *t* tests revealed a significantly higher FC over time in the PP group (mean = 0.1, sd = 0.109, t(55) = 6.866, p < 0.001) but no change over time in the MC group (mean = − 0.038, sd = 0.15, t(52) = − 1.834, p = 0.072) for this cluster. Baseline analyses revealed a negative correlation between these regions (mean = − 0.078, sd = 0.11), revealing a shift from negative to positive correlation for the PP group.

The second significant group difference (p_FWE_ = 0.001, k = 288, T = 4.88) was found between the **right** HG and a cluster in the right precentral gyrus, extending into right superior frontal gyrus, and right middle frontal gyrus. The Human Motor Area Template (HMAT)^74^, was used to further assess the exact sensorimotor regions of this cluster. It showed that it is primarily located in the right dorsal premotor cortex (dPMC), but also the right ventral premotor cortex (vPMC). These subdivisions are not included in the AAL3 atlas. Post-hoc *t* tests further revealed higher FC after twelve months for the PP group (mean = 0.09, sd = 0.127, t(55) = 5.4, p < 0.001), but no significant Bonferroni-corrected change in the MC group (mean = − 0.039, sd = 0.13, t(52) = − 2.204, p = 0.032). These regions were already positively correlated at baseline (mean = 0.084, sd = 0.12).

The last significant group difference was found between the **right** HG and a cluster with peak activity in the right supramarginal gyrus (p_FWE_ < 0.001, k = 360, T = 4.35). Post-hoc tests showed no change in the MC group (mean = − 0.038, sd = 0.145, t(52) = − 1.917, p = 0.061) and a significant FC increase in the PP group (mean = 0.094, sd = 0.146, t(55) = 4.805, p < 0.001). These regions were also positively correlated at baseline (mean = 0.062, sd = 0.14).

#### Right motor hand area—T2 results, PP > MC

**Figure 4:**
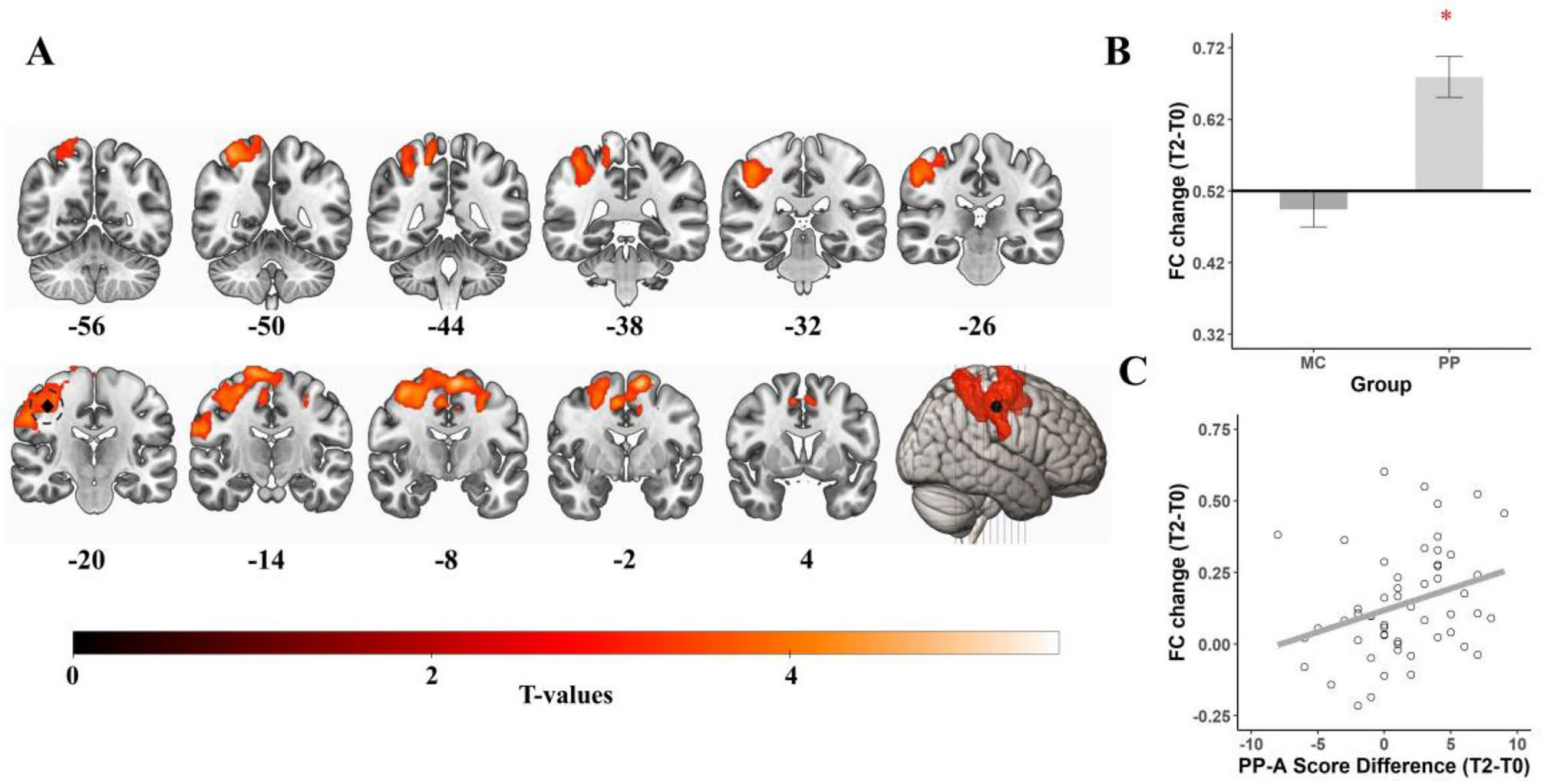
Results for right M1H seed. Regions that show a significant PP > MC group difference after 12 months of interventions (**A**). Post-hoc *t* tests revealed that only the PP group showed a significant Bonferroni-corrected difference to 0, indicating a FC change over the 12 months of training in the PP group and no change in FC for the MC group (**B**). The mean values for each group are displayed in relation to the baseline correlation. The bold line represents the baseline correlation. Further, the FC change in the PP group showed a significant positive correlation with PT-A score differences after 12 months, which is linked to fine motor finger dexterity (**C**). Error bars represent the standard mean error. Red asterisk (*) depict significant post-hoc *t* tests. *MC* music listening/musical culture group, *PP* piano practice group, *FC* functional connectivity, *T0* baseline measurement, *T2* 12 months measurement, *PT-A* Purdue Pegboard test assembly condition. The seed location is shown in black and circled. Coronal slices are displayed in radiological convention (left = right hemisphere, right = left hemisphere).

Analyses revealed significant group differences after 12 months of interventions for FC between the **right** M1H and a large cluster (p_FWE_ < 0.001, k = 2880, T = 5.31, Fig. 4A) covering the right postcentral gyrus, right precentral gyrus, right superior frontal gyrus (dPMC), as well as right and left supplementary motor areas (SMA). Post-hoc *t* tests (Fig. 4B) revealed a significant increase in FC between the right M1H and this cluster in the PP group (mean = 0.159, sd = 0.212, t(55) = 5.61, p < 0.001), but no significant change in the MC group (mean = − 0.026, sd = 0.179, t(52) = − 1.04, p = 0.303). Baseline testing revealed a high positive correlation between these regions (mean = 0.515, sd = 0.16).

#### Left hippocampal formation—T2 results, PP > MC

**Figure 5:**
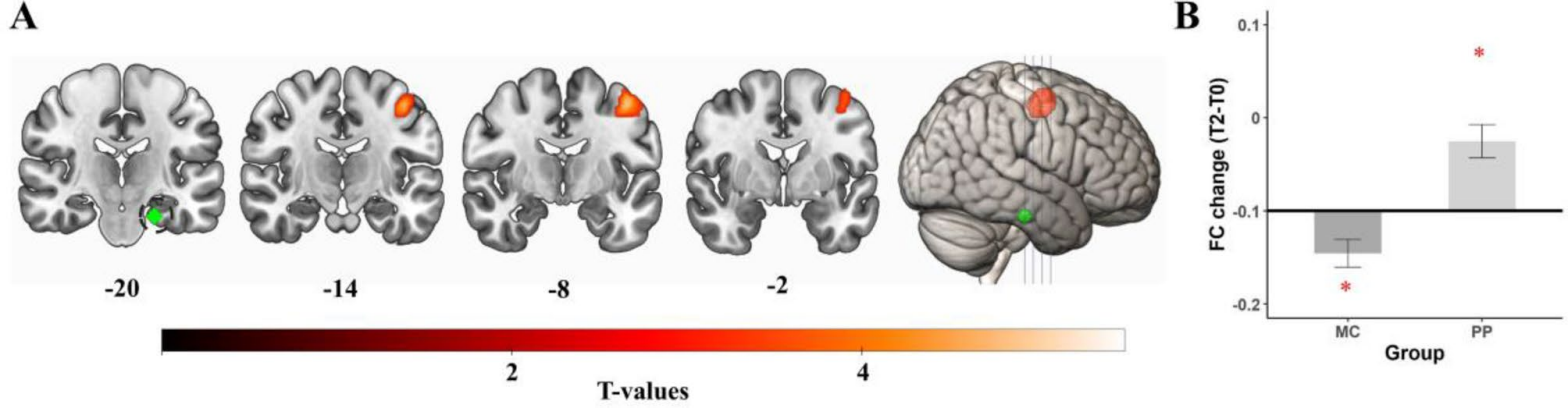
Results for left HPF seed. Regions that show a significant PP > MC group difference after 12 months of interventions (**A**). Post-hoc t-tests revealed that both groups showed significant Bonferroni-corrected differences to 0, indicating a significant FC increase over the 12 months of training in the PP group and a significant FC decrease for the MC group (**B**). The mean values for each group are displayed in relation to the baseline correlation as bar plots. Bold line represents the baseline correlation. Error bars represent the standard mean error. Red asterisk (*) depict significant post-hoc t-tests. *MC* music listening/musical culture group, *PP* piano practice group, *FC* functional connectivity, *T0* baseline measurement, *T2* 12 months measurement. The seed location is shown in green and circled. Coronal slices are displayed in radiological convention (left = right hemisphere, right = left hemisphere).

After twelve months of interventions, a significant effect of group was detected between the **left** HPF and the left pre-and postcentral gyrus (p_FWE_ = 0.006, k = 208, T = 5.32). Post-hoc tests revealed a significant decline in FC for the MC group (mean = − 0.045, sd = 0.108, t(52) = − 3.054, p = 0.004) and a significant FC increase for the PP group (mean = 0.075, sd = 0.134, t(55) = 4.16, p < 0.001, see Fig. 5). Further analysis revealed an anticorrelation at baseline (mean = − 0.102, sd = 0.10), resulting in a higher anticorrelation of the MC group and a decreased anticorrelation of the PP group over time.

### Behavioral measures and correlational analyses

As the FC analyses only revealed significant results after 12 months, we also only analyzed the corresponding behavioral tests after 12 months.

#### Midi-based scale analysis—T2 results

Mixed-design ANOVA revealed a significant main effect of time [F(1, 95) = 57.331, p < 0.001] and a time*group interaction [F(1, 95) = 9.559, p = 0.003], but no significant main effect of group (p = 0.272) after 12 months of interventions. Post-hoc *t* tests revealed no significant difference between groups at T0 [t(95) = 0.560, p = 0.577], but a significantly lower mean standard deviation from the IOI for PP at T2 [t(95) = 3.934, p < 0.001]. Correlational analyses did not reveal any significant connection between MSA and FC changes. This indicates that the PP group showed a higher improvement than the MC group, with no link to any significant FC changes.

#### Purdue Pegboard test—T2 results

The PT consists of four test scores. Mixed-design ANOVA in the analysed sample, including baseline and 12 months data revealed a significant effect of time for PT–RH [F(1, 107) = 13.584, p < 0.001] as well as a significant time*group interaction [F(1, 107) = 4.184, p = 0.043]. Post-hoc *t* tests revealed no significant difference between groups at T0 [t(107) = 0.287, p = 0.775], but a significantly higher PT–RH score for PP at T2 [t(107) = 2.110, p = 0.037]. Further, post-hoc tests only revealed a significant change over time for PP [t(55) = 4.037, p < 0.001], but not for MC [t(52) = 1.167, p = 0.249]. Analyses for the PT-LH revealed the same effects: a significant effect of time [F(1, 107) = 10.112, p = 0.002] and a significant time*group interaction [F(1, 107) = 5.734, p = 0.018]. Post-hoc *t* tests revealed no significant difference between groups at T0 [t(107) = 0.063, p = 0.949], but a significantly higher PT-LH score for PP at T2 [t(107) = 2.130, p = 0.035]. Post-hoc t-tests only revealed a significant change over time for PP [t(55) = 3.96, p < 0.001], but not for MC [t(52) = 0.553, p = 0.582]. This indicates that the significant effect of time in the mixed-design ANOVA is driven by significant improvements of the PP group in PT–RH and PT-LH, but no improvements of the MC group.

No significant effect was found for PT-BH. Analyses of PT-A revealed no significant effect of time, but a significant time*group interaction [F(1, 107) = 4.968, p = 0.028]. Post-hoc *t* test for PT-A revealed a significant increase over time in the PP group [t(55) = 3.117, p = 0.003], but no change over time in the MC group [t(52) = 0.385, p = 0.702]. No significant main effect of group occurred in any of the analyses.

As the FC analysis revealed significant group differences between the right M1H seed and other bilateral motor related areas we correlated these changes with changes in PT-BH and PT-A to investigate whether they are related to gross motor movements and/or fine motor dexterity in the PP group. Correlational analyses revealed a significant correlation between changes related to the right M1H seed after 12 months of training and the PT-A change score (T2-T0): r = 0.371, p = 0.005 (this result survives Bonferroni correction, Fig. 4C). There was no significant correlation with the PT-BH score difference (p = 0.64). This indicates that the increased FC between the right M1H and other bilateral motor-related regions is related to increases in finger/ fingertip dexterity rather than gross motor movement.

## Discussion

Although the literature on brain-related effects of making music is extensive and some initial studies show positive effects of learning to play an instrument on cognitive functioning in healthy older adults, large-scale RCTs are still rare. This is where the current study sets in. We investigated the effects of piano training on FC in a population of healthy older adults as compared to an active control group participating in musical culture lessons. While six months of training did not lead to any significant changes in seed-based FC, twelve months of piano training led to a wide range of FC changes in the piano group. These include a higher FC between bilateral motor-related areas, which was significantly correlated with improvements in fine motor dexterity. In addition, participants who learned to play the piano showed higher coupling between right-lateralized regions involved in auditory-motor integration. This result is complemented by a higher anticorrelation between the right HG and dPCC, which can be interpreted as a higher attentional capacity for auditory stimuli. Although PP led to a measurable increase in piano playing abilities, these changes could not be related to any FC changes. A reason might be that piano playing abilities were tested in the right hand while FC changes were found for the right motor cortex, responsible for the left hand.

Learning to play an instrument includes learning to initiate and coordinate precise and timely (hand) movements based on acoustic feedback. To achieve this, coupling of auditory and motor regions is necessary^76^. In the current study, learning to play the piano resulted in a higher FC between right HG and right dPMC and vPMC after 12 months of training. Both regions are thought to have a role in sensory-motor transformations^1,77^. While the dPMC is involved in motor planning and the selection of movement parameters based on sensory information, and therefore has a more indirect role in sensory-motor transformations, the vPMC guides movements in response to a sound^1,78^.

A higher FC between right HG and right vPMC was previously found in musicians compared to non-musicians^12^. However, in the current study, the cluster is predominantly located in the dPMC (this region was not investigated in the other study). It is possible that after only one year of training in an older adult population, playing the piano still relies a lot on planning and selecting the correct movements and not on automatic processes as in professional musicians. This might explain the higher coupling between the right HG and the dPMC and not the vPMC. This observation corroborates with findings from a meta-analysis^79^, that found the dPMC to be one of the main structures to underlie motor learning. Further, the right dPMC is essential for rhythmic auditory-motor synchronization^80^.

Auditory and premotor regions are connected via the dorsal auditory stream, which further includes inferior parietal and prefrontal regions and is involved in auditory-motor integration^77,81,82^. Anatomically these regions are connected by the arcuate fasciculus^39^. Especially the right arcuate fasciculus has been linked to musical auditory-motor feedback^83–85^. Vaquero et al.^84^ for example could link right arcuate fasciculus integrity and volume to learning speed of melodies in non-musicians and therefore demonstrate a right-lateralized integration of the dorsal auditory stream for music. In addition, the arcuate fasciculus arches over the inferior parietal lobule, which encompasses the supramarginal gyrus, where increased FC with the HG after 12 months of piano training was found in this study. This further supports the claim of higher coupling between dorsal auditory stream regions induced by piano training.

Therefore, in summary, we found specific right-lateralized FC improvements in the dorsal auditory processing stream in areas structurally connected by the arcuate fasciculus induced by piano training but no changes in the left hemisphere. These results speak in favor of a right lateralization for musical processing and learning^84,86–88^ and show that even in older adults, audio-motor training can lead to profound changes in the underlying networks.

The largest significant cluster was found between the right M1H seed and regions primarily belonging to the SMN, namely ipsilateral pre- and postcentral gyrus, dPMC, as well as bilateral SMAs^30,89^ in participants who learned to play the piano. The precentral gyrus, contains the primary motor cortex and is associated with the execution of movements in general, while the postcentral gyrus contains the primary somatosensory cortex and sends somatosensory feedback, necessary for movement coordination and timing, to the primary motor cortex^90^. The SMA is thought to play a vital role in the set-up and execution of action plans of voluntary, self-initiated movements. With respect to music, it has been associated with the processing of temporal aspects such as rhythm and beat, but also motor control in music production and musical mental imagery^91^. In addition, the SMA is thought to function as an error detector ^92^. All of these areas have previously been identified as being essential for motor skill learning^79^.

Interestingly, only the right, but not the left M1H seed showed an increased FC with other motor-related areas. Since all participants were right-handed, learning to play the piano, promotes the use of the otherwise less recruited left hand. It is, therefore, possible that the right M1H, responsible for the weaker left hand, may have more room for improvement and therefore shows higher FC changes with surrounded areas after 12 months of training. This might also explain why no significant FC changes were visible after six months of training, as the left hand was more extensively trained in the second semester of training. This matches the PT results, where PT-LH improved more from month 6 to 12 than from month 1 to 6^45^. Behaviorally, these FC changes were related to improvements in PT-A. It is plausible that right hand fine motor control was more developed in our study population as it is more extensively used in daily life, and therefore this improvement in bimanual fine motor dexterity is primarily driven by improvements in left hand fine motor function. However, the PT did not reveal left-hand advantages. This finding is supported by a study of Hyde and colleagues^93^. They investigated the effects of 15 months of piano training in children aged 6 to 7 years and showed as a prominent result an increase of gray matter in the right but not in the left M1H. These changes were significantly correlated with left hand fine finger motor skills^93^.

In summary, piano training seems to increase the integration between sensorimotor areas involved in the planning, execution, and feedback-control/error correction of complex, fine-tuned and temporally coordinated movements needed for playing the piano. It is plausible that learning to master a demanding activity like playing the piano requires and enhances communication among relevant areas even in older adults.

The most prominent resting-state network, the DMN, is deemed a task-negative network, as it is more active during rest than during task performance, and is anticorrelated with task-positive networks^94,95^. In theory, the higher the anticorrelation, the easier it is to switch between the networks, as they do not interfere with each other during tasks. Therefore, the magnitude of this anticorrelation between the DMN and higher-order cognitive networks has been associated with a more efficient task performance^96^.

In the current study, an increased anticorrelation was found between the right HG seed and the dPCC in the PP group after a full year of training. The dPCC has not only been been shown to be one of the key regions within the DMN, but also to task-related networks, including an executive, motor, and sensory network^97^. In this context, Leech and Sharp^72^ propose that the dPCC controls the conversion between internally and externally guided attention. Thus, it is interesting that in the current study we found a higher anticorrelation with this area, while piano learning elicits increased attention on auditory stimuli. This higher anticorrelation between the dPCC and the right HG may be interpreted as a better capacity to focus the attention on external auditory stimuli after one year of piano training. At the same time we found a shift from a negative to positive correlation in the PP group between right HG and left superior parietal lobule, a region that has been associated with motor attention^98^. During piano playing these regions need to interact more closely which could explain the shift in correlation.

At last, we also found a reduced anticorrelation between the left HPF and ipsilateral primary motor and somatosensory cortices, close to the left M1H seed in the PP group. Traditionally, the HPF is thought to be involved in episodic memory, and is not only associated with explicit but also implicit motor learning^99^. During piano learning, it is important to store motor programs into memory. One can speculate that this led to more co-activations between the networks, which could explain this observed reduced anticorrelation. Further, the MC group showed a higher anticorrelation between these systems. As this group did not participate in any motor training but still engaged in theoretical learning, this could have led to a higher independence of the memory system.

The main strength of the current study is the large sample size of 109 analyzed subjects, which is rare for RCTs involving musical activities and to our knowledge to date the largest study investigating effects of musical training in healthy older adults’ FC at rest. On the one hand, the use of an active control group poses a great advantage, as it enabled us to investigate effects specific to the learning of a new skill; i.e., playing the piano, and differentiate it from effects induced by bare social interaction and engaging in a new activity. On the other hand, it did not allow us to investigate how FC would have developed in community-dwelling older adults and whether participating in MC lessons already has some positive effects. This is also a reason why it is not possible to meaningfully interpret the significant effect of time between the right IFG and the left angular gyrus, as this change could be induced by the common point between both interventions, musical activities, or by normal aging.

In addition, the use of seed-based FC analysis also comes with advantages and disadvantages. While it is possible to investigate specific hypotheses for specific seed regions, this also limits the analyses and possible effects in other regions. This bias on seed selection also makes it difficult to compare the current study with previous studies investigating the effect of musical training on FC or FC differences between musicians and non-musicians. Most of these studies used different seed regions and did not investigate seed-to-whole-brain FC, but seed-to-seed FC, which might also explain some of the different outcomes. However, this is a general problem of seed-based rs-fMRI analyses as it highly depends on the selected seeds, which can lead to high variability in results between studies and therefore interpretation^100^.

The heterogeneity between study designs and their analysis is a known problem in the field of aging research and longitudinal aging research in particular, which is in depth discussed by Oschwald et al.^101^ on the example of the interaction of brain structure and cognitive abilities in healthy older individuals. One interesting approach to analyze longitudinal data proposed in this review are latent change score models. This type of statistical model enables to look at measurement invariance across time points and taking individual differences into account^101^. It has already been used in another paper based on the same study cohort as the current paper using structural brain data^45^, but it would be interesting to use this type of statistical modelling on the rs-FC data as well in the future.

In our study, we didn't gather ethnicity data. All participants were native German or French speakers, therefore mostly Caucasian, with non-Caucasian individuals growing up in Western environments. In France, where half of the Geneva participants originated, collecting ethnicity data is legally prohibited for equal treatment reasons.

Some studies note differences in FC of the DMN in elderly from different ethnic backgrounds^102,103^, as well as ethnic differences in the likelihood of developing dementia^104^, thus the current findings might not be generalizable to the global population. This poses an interesting and relevant research gap that should be explored specifically in future research.

In conclusion, the current study demonstrated piano training-induced FC changes in specific sensorimotor and auditory-motor networks in healthy older adults after 12 months of training. Although evidence for the positive effect of learning to play the piano to counteract or slow down age-related decline is limited in the current study, the fact that the older adult brain shows neuroplastic adaption to a newly learned skill raises hope that it is possible to actively influence the course of neurological aging, potentially by increasing cognitive reserve. Therefore, future studies are needed to see whether learning to play the piano at an advanced age might be an effective tool to reduce the burdens related to age-related cognitive decline and sensorimotor deterioration in the long term. That music practice might be effective is plausible, as musical activities involve several functions and skills that are prone to decline with age, such as working memory, attention, processing speed, executive functions, and even abstract reasoning. The fact that a majority of individuals experiences music-making as pleasant would allow long lasting involvement after retirement. Overall, the current study poses an essential starting point showing that functional neuroplasticity in older adults is possible in response to musical training.

## Data Availability

The datasets generated during and/or analyzed during the current study are available from the corresponding author on reasonable request.
